# Surface Potential-Controlled Oscillation in FET-Based Biosensors

**DOI:** 10.3390/s21061939

**Published:** 2021-03-10

**Authors:** Ji Hyun Kim, Seong Jun Park, Jin-Woo Han, Jae-Hyuk Ahn

**Affiliations:** 1 Department of Electronic Engineering, Kwangwoon University, Seoul 01897, Korea; jayyullmoo@naver.com (J.H.K.); sjun96@naver.com (S.J.P.); 2 Center for Nanotechnology, NASA Ames Research Center, Mountain View, CA 94035, USA; jin-woo.han@nasa.gov; 3 Department of Electronics Engineering, Chungnam National University, Daejeon 34134, Korea

**Keywords:** field-effect transistor, chemical and biological sensor, pH sensor, surface potential, oscillation frequency, ring oscillator, extended gate

## Abstract

Field-effect transistor (FET)-based biosensors have garnered significant attention for their label-free electrical detection of charged biomolecules. Whereas conventional output parameters such as threshold voltage and channel current have been widely used for the detection and quantitation of analytes of interest, they require bulky instruments and specialized readout circuits, which often limit point-of-care testing applications. In this study, we demonstrate a simple conversion method that transforms the surface potential into an oscillating signal as an output of the FET-based biosensor. The oscillation frequency is proposed as a parameter for FET-based biosensors owing to its intrinsic advantages of simple and compact implementation of readout circuits as well as high compatibility with neuromorphic applications. An extended-gate biosensor comprising an Al_2_O_3_-deposited sensing electrode and a readout transistor is connected to a ring oscillator that generates surface potential-controlled oscillation for pH sensing. Electrical measurement of the oscillation frequency as a function of pH reveals that the oscillation frequency can be used as a sensitive and reliable output parameter in FET-based biosensors for the detection of chemical and biological species. We confirmed that the oscillation frequency is directly correlated with the threshold voltage. For signal amplification, the effects of circuit parameters on pH sensitivity are investigated using different methods, including electrical measurements, analytical calculations, and circuit simulations. An Arduino board to measure the oscillation frequency is integrated with the proposed sensor to enable portable and real-time pH measurement for point-of-care testing applications.

## 1. Introduction

Field-effect transistor (FET)-based biosensors have received significant attention for their label-free electrical detection of chemical and biological species. Because of their inherent similarity with conventional FETs in terms of device structure and fabrication process, FET-based biosensors offer the well-known advantages of modern electronic devices such as high-density integration of sensor arrays in a compact size, reliable fabrication with high reproducibility, and low-cost mass fabrication. The replacement of the solid-state gate of a conventional FET to the liquid–solid interface allows an FET-based biosensor to have an open surface for detecting chemical reactions or biological binding of interest. Since the invention of ion-sensitive field-effect transistors that facilitate the detection of ions by P. Bergveld in 1970 [1], FET-based biosensors have been widely utilized for detecting antibody–antigen [2,3,4], DNA [5,6], and viruses [7,8,9] for disease diagnosis, basic research, and point-of-care testing (POCT). Advanced nanotechnology and the discovery of new materials have improved sensing performances such as sensitivity and limit of detection by the incorporation of silicon nanowires [10,11,12], carbon nanotubes [13,14], graphene [15,16,17], two-dimensional transition metal dichalcogenide [18,19], and metal oxide nanostructures [20,21,22] into basic elements of FET-based biosensors.

An FET-based biosensor transduces a binding event occurring on the gate surface into a change in the electrical characteristics, such as the threshold voltage or channel current [23,24,25]. For example, the reversible binding of hydrogen ions on the active sites of a gate (i.e., ion-sensitive membrane) results in a change in the surface potential of the channel; this phenomenon can be utilized in developing pH sensors [26,27]. When a receptor functionalized on an FET surface captures its analyte, the intrinsic charge of the analyte changes the surface potential of the FET. This mechanism enables label-free electrical detection of antibody-antigen binding and DNA binding [5,10,11]. Binding-induced conformational changes (i.e., aptamers) result in changes in the local potential near the FET surface [28,29,30]. The existence and quantity of analytes in a test solution are correlated with the amount of change in the threshold voltage or channel current. Hence, the threshold voltage and channel current have been widely used as output parameters for FET-based biosensors.

Among several methods for monitoring the threshold voltage and channel current, a parameter analyzer, which is a specialized equipment for characterizing the electrical properties of FET devices, is widely used for extracting output parameters owing to its precision measurement with high resolution [31,32,33]. However, its bulky size and exorbitant cost limit POCT applications. As an alternative for POCT applications, readout circuits for monitoring output parameters have been constructed in the form of small-sized integrated circuits (ICs) by using modern electronics technology [34,35]. Despite the portability of ICs, their customized fabrication and high cost in design and development limit their generic use in readout circuits.

The sensing process outputs analog values of both voltage and current readings. Subsequently, these analog values are digitalized using a readout circuit. Traditionally, the analog output is input into an analog-to-digital converter (ADC) to interface with digital electronics. This is associated with the fact that the traditional computer architecture, i.e., the von Neumann architecture, has long evolved digitally to countermeasure noise immunity. Recently, the neuromorphic architecture has attracted significant attention in mimicking the neurobiological structure of the nerve system [36,37,38,39,40]. One of the neuromorphic architectures is the spiking-neural network [41,42], which mimics the natural neural network the most. When the traditional ADC is integrated with the neuromorphic architecture, the ADC levels must be converted into spiking rates, thereby resulting in circuit inefficiency, propagation delay, and power consumption. Therefore, an alternative method that is the most compatible with spiking neural networks must be identified.

In this study, we demonstrate a signal conversion scheme using frequency as a new output parameter of FET-based biosensors to complement the conventional output parameters. The simple connection of a ring oscillator to a readout transistor enables the conversion from a surface potential to an oscillation signal. The oscillation frequency can be regarded as the spiking rate of the neural network. Hence, the frequency and spiking rate can be used interchangeably. The spiking rate as an output can be input into the axillary spiking neural network. To demonstrate the spiking rate altered by biosensing, in this study, the oscillation frequency is measured using a portable, general-purpose microcontroller (i.e., Arduino) by counting the number of peaks in a given time. Electrical measurement of the linear correlation between the oscillation frequency and pH value confirms that the oscillation frequency is a suitable output parameter for FET-based biosensors. We discuss the change in the pH sensitivity of the oscillation frequency based on experiments, analytical calculations, and circuit simulations. Finally, we present the future prospects of using the oscillation frequency in portable sensing systems based on real-time pH measurements using an Arduino board operated via open source codes.

## 2. Experimental Section

### 2.1. Fabrication of Sensing Electrodes

The fabrication of pH-sensitive electrodes was based on our previous study [9,43]. Briefly, a 15-nm-thick Al_2_O_3_ layer was deposited on metal electrodes (Cr/Au = 3 nm/100 nm) via electron beam evaporation. The dimensions of the metal electrodes were 3 mm × 3 mm or 5 mm × 5 mm. A reservoir to fill the test solution was constructed on the Al_2_O_3_ sensing layer using a silicone elastomer (Kwik-cast, World Precision Instruments, Inc., Sarasota, FL, USA). Optical microscopy images of the fabricated sensing electrodes are presented in Supplementary Materials (Figure S1).

### 2.2. Construction of Frequency Measurement System

**Figure 1:**
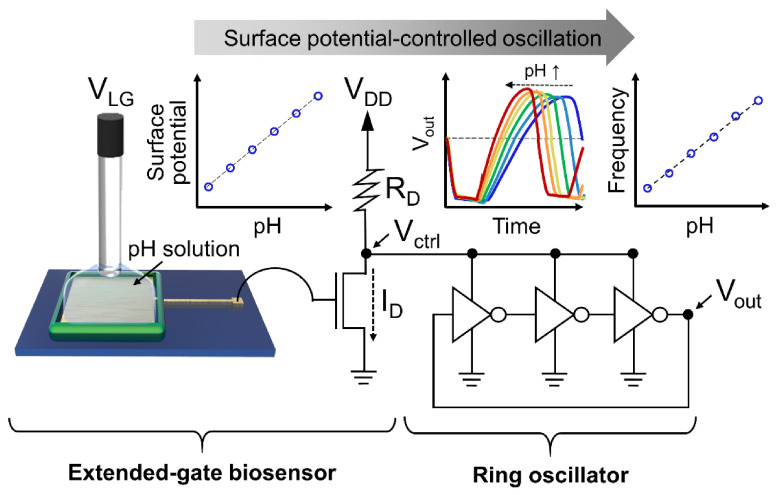
Schematic illustration of surface potential-controlled oscillation. An extended gate (i.e., sensing electrode) that generates surface potential based on pH is connected to a readout transistor, where surface potential is converted to drain current (*I_D_*). Subsequently, *I_D_* that flows through the load resistor (*R_D_*) determines the control voltage (*V_ctrl_*), which is input to the ring oscillator that generates an oscillation signal. Oscillation frequency is utilized as an output parameter for pH sensing.

A schematic illustration of the experimental setup is shown in Figure 1. A pH buffer solution was added to the reservoir constructed on the sensing electrode, and an Ag/AgCl reference electrode (MF-2052, Bioanalytical Systems, Inc., West Lafayette, IN, USA) was immersed in the test solution. The probing pad of the fabricated sensing electrode was connected to the gate terminal of a commercially available n-type FET (CD4007UBE, Texas Instruments, Dallas, TX, USA), which was used as a readout transistor to convert the surface potential of the sensing electrode to the drain current (*I_D_*). This configuration is known as the extended-gate FET [44]. The control voltage (*V_ctrl_*) terminal of a three-stage ring oscillator (CD4007UBE, Texas Instruments, Dallas, TX, USA) was connected to the drain terminal of the readout transistor, where the load resistor was connected. Two separate CD4007UBE devices were used to construct the readout transistor and three-stage ring oscillator. The ring oscillator arrangement using CD4007UBE devices is provided in Supplementary Materials (Figure S7b).

### 2.3. Electrical Characterization

A liquid-gate voltage (*V_LG_*) was applied to the reference electrode, and a supply voltage (*V_DD_*) was applied to the load resistor connected to the drain terminal of the readout transistor using with a SourceMeter (Model 2614B, Keithley Instruments, Inc., Cleveland, OH, USA). The voltage drop in the load resistor determined the *V_ctrl_* input to the ring oscillator. The oscillation frequency of the ring oscillator was measured at the output terminal of the ring oscillator using an oscilloscope (EDUX1002A, Keysight Technologies, Santa Rosa, CA, USA). The threshold voltage (*V_T_*), which is defined as the gate voltage that flows a *I_D_* of 100 nA in the subthreshold region, was extracted using the SourceMeter by sweeping the *V_LG_* and measuring the *I_D_* after removing the load resistor and ring oscillator.

### 2.4. Portable Sensing System

An Arduino board (Arduino Uno R3, Arduino LLC, Somerville, MA, USA), which is a general-purpose microcontroller, was used to construct a portable sensing system to measure the oscillation frequency. A liquid crystal display (LCD) module (SZH-EK101, SMG, China) was connected to an Arduino board to display the measured frequency. All components including the sensing electrode, readout transistor, three-stage ring oscillator, Arduino board, and LCD module were integrated on a printed circuit board (PCB). A 9 V battery was used to supply power to each component. The detailed Arduino board-pin mapping and the applied voltages are shown in Figure S7. The Arduino source codes that counted the number of oscillations were uploaded to the Arduino board where the LCD was connected to display the oscillation frequency according to the pH. The Arduino source codes for the frequency measurement are provided in Supplementary Materials.

## 3. Results and Discussion

### 3.1. Operation Principle: Surface Potential-Controlled Oscillation

Compared with conventional FET-based biosensors, a distinctive feature of the proposed biosensor is the parameter conversion scheme constructed using a ring oscillator that converts the *V_T_* or *I_D_* to the oscillation frequency (Figure 1). Although we used a three-stage ring oscillator, it was not necessarily the best option. The number of stages allows the system designer to select the spiking rate of interest. Alternatively, increasing the supply voltage at a specified oscillator can be a method to increase the nominal point of the spiking rate. As the odd number of stages increases, the oscillation frequency can be decreased. The typical frequency range can vary from low frequency (~20 Hz), audio frequency (20 Hz–20 kHz), and up to radio frequency (100 kHz–100 GHz). Furthermore, other types of oscillators such as the resistor-capacitor or inductor-capacitor, or variations of the ring oscillator can be used as requred. The oscillation frequency can be measured using not only a bulky oscilloscope, but also using a compact, portable general-purpose microprocessor (i.e., Arduino board); this will be discussed later.

The operating mechanisms of extended-gate biosensors have been investigated previously [24]. A change in the surface potential of the sensing electrode upon the binding of chemical and biological species results in a change in the threshold voltage, consequently modulating the *I_D_* of the readout transistor. The new components, i.e., the ring oscillator and load resistor, incorporated in the output of the extended-gate biosensor convert the *I_D_* to the oscillation frequency. The amount of *I_D_* flowing through the load resistor determines the voltage at the drain terminal (i.e., *V_ctrl_*), which is the supply voltage of the subsequent ring oscillator where the oscillation frequency is changed by the value of *V_ctrl_*. In the entire procedure, the surface potential of the sensing electrode results in an oscillation frequency, indicating that the frequency can be utilized as a new output parameter for FET-based biosensors.

### 3.2. pH Sensing Characteristics

**Figure 2:**
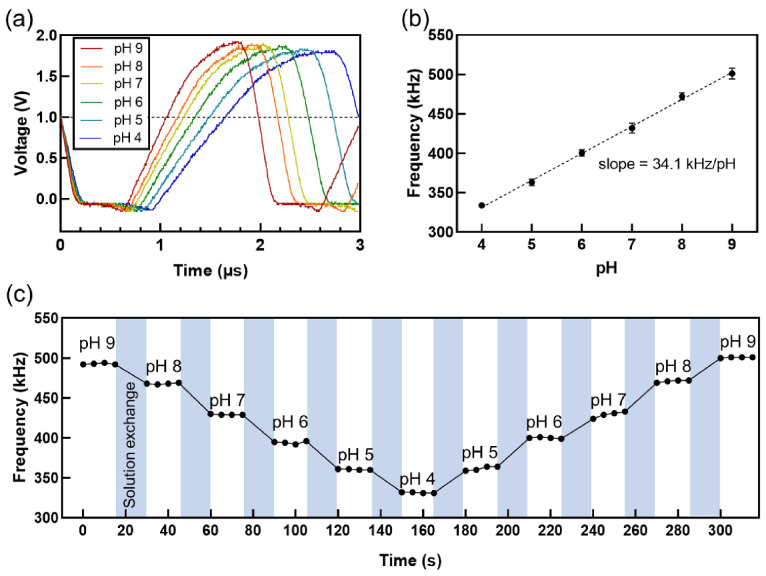
pH sensing characteristics. (**a**) Output waveform of ring oscillator as a function of pH. Starting point of each curve overlapped at 0 s. Dotted line provides visual guidance of shift in period (inverse of frequency) due to pH. (**b**) Oscillation frequency vs. pH, showing linear slope of 34.1 kHz/pH. (**c**) Time-resolved pH measurement. pH sensing results confirmed ability of proposed sensor in detecting changes in surface potential.

We investigated the relationship between the oscillation frequency of the proposed sensor and the pH value of the test solution to verify the feasibility of the oscillation frequency as an output parameter for FET-based biosensors. The pH sensing experiment is a popular proof-of-concept example that confirms the ability of the newly designed sensor in detecting charged biomolecules [9,10,43]. With an increase in the pH value of the buffer solution, the output signal, which is the output voltage as a function of time, shifted to the left, thereby increasing the oscillation frequency (Figure 2a,b). The frequency depended linearly on the pH value, with high reproducibility (Figure 2b,c).

Our additional experiments revealed that the oscillation frequency was directly correlated with *V_T_*. The same sensing electrode used to obtain Figure 2a,b shows a similar trend in terms of the linear dependence of *V_T_* on pH (Figure S2). The pH-induced *V_T_* shift can be explained by site-binding theory, in which hydrogen ions reversibly bind to active OH sites on the Al_2_O_3_ surface, thereby generating a surface potential based on pH [26,45]. When a sensing electrode indicates a nonlinear dependence of *V_T_* on pH due to undesired defects on the surface, the oscillation frequency as a function of pH exhibits the same nonlinear trend, which does not facilitate correct measurements (Figure S3). This result implies that the preparation of sensing electrodes that generate sensitive and reliable surface potentials is crucial in the design of high-performance surface potential-controlled oscillations.

Further measurements indicate that *V_T_* modulated the oscillation frequency. The oscillation frequency of the ring oscillator was proportional to the gate voltage (*V_G_*) of the readout transistor via the transfer of *V_G_* into *V_ctrl_* (Figure S4). It is noteworthy that changing *V_G_* in these measurements can be considered as a pH-induced *V_T_* shift because *V_T_* is defined as the effective *V_G_* that flows a constant *I_D_*. The close relationship between *V_T_* and oscillation frequency allows the frequency to be a versatile output parameter without sacrificing the advantages of *V_T_* in FET-based biosensors.

Although we simply measured a small pH range of 4 to 9 to demonstrate surface potential-controlled oscillation, the Al_2_O_3_-deposited sensing electrode can detect a wider pH range of 3 to 11, as confirmed by the pH sensing characteristics of our previous study [46]. The maximum *V_T_* shift (i.e., voltage signal) is measured as ~0.4 V from pH 3 to 11. A curve for the oscillation frequency as a function of the gate voltage (Figure S4d) shows that an input signal larger than 0.5 V can be linearly converted to the oscillation frequency. Because the allowed input voltage for linear frequency conversion is larger than the maximum voltage signal generated from the sensing electrode, our sensor is expected to measure the pH response of the Al_2_O_3_ surface over a pH range of 3 to 11.

It is expected that the pH sensitivity depends on the temperature because the surface potential ($\psi_{o}$) of the sensing electrode can be described with the Nernst equation [26]:

$$\psi_{o}=2.303\frac{kT}{q}\log[a_{H^{+}}/a_{H^{+}}{}^{o}]$$

where *k* is the Boltzmann constant, *T* is the absolute temperature, *q* is the elementary charge, $a_{H^{+}}$ is the solution’s ion activity, and $a_{H^{+}}{}^{o}$ is the ion activity at the point of zero charge. In our previous study, the Al_2_O_3_-deposited electrode generated 43.7 mV/pH at 25 °C (298 K) and 45.4 mV/pH at 40 °C (313 K), where the pH sensitivity improvement by 4% is similar to the Nernst equation-derived calculation by 5% [46]. A similar temperature dependence on pH sensitivity has been reported elsewhere [27]. This temperature instability should be further compensated in a readout circuit by measuring the test solution temperature.

It is possible to detect biomolecular interactions in real time if the sensing electrode is functionalized with receptors to capture target biomolecules. However, nonspecific binding of interfering biomolecules present in serum and blood generates noises to deteriorate detection sensitivity. To prevent nonspecific binding on the sensing electrode, the remaining binding sites on the surface are generally blocked by a bovine serum albumin (BSA) solution [32,47]. In addition, tailoring the sensor surface with a polymer polyethylene glycol (PEG) can further reduce nonspecific signals from interfering biomolecules owing to the hydrophilic PEG on the surface, which enables real-time immunodetection in whole serum [47,48].

### 3.3. Signal Amplification

Understanding the effects of circuit parameters on the frequency signal is important when establishihng methods for signal amplification. Unlike the *V_T_* signal, which requires dual gates for siginal amplificaiton [12,25,49,50], the single-gate configuration is sufficient for increasing the frequency sensitivity in units of Hz/pH by controlling the values of other circuit parameters, such as the number of stages (*N*), load resistance (*R_D_*), and *V_LG_*.

**Figure 3:**
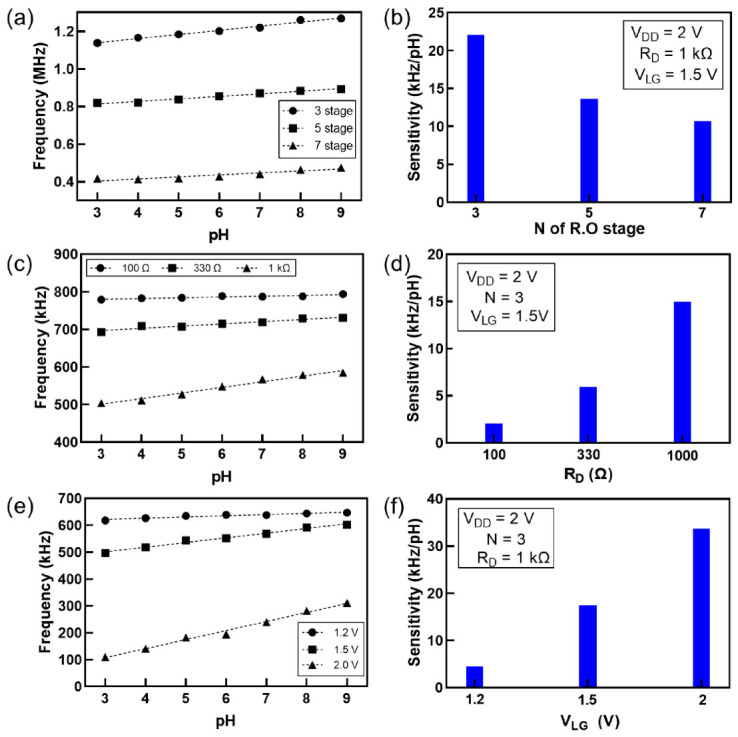
Effects of circuit parameters on sensitivity. (**a**) Oscillation frequency vs. pH for different numbers of stages (*N*). (**b**) Dependence of sensitivity on *N*. (**c**) Oscillation frequency vs. pH for different values of load resistance (*R_D_*). (**d**) Dependence of sensitivity on *R_D_*. (**e**) Oscillation frequency vs. pH for different values of liquid-gate voltage (*V_LG_*). (**f**) Dependence of sensitivity on *V_LG_*.

Figure 3a shows the frequency as a function of pH for *N* = 3, 5, and 7. The sensitivity increased as *N* decreased (Figure 3b). The frequency of the ring oscillator (*f*) can be modeled as *f = 1 / (2Nt_p_)*, where *t_p_* is the propagation delay time per stage. Let us assume that the per-stage delay change from the initial value is Δ*t_p_* = *t_p_* − *t_p,i_* at a given pH. Therefore, the frequency change, i.e., the sensitivity, Δ*f* = –Δ*t_p_* / (2*Nt_p_t_p,i_*). Because *t_p_* and *t_p,i_* are independent of *N*, the simple frequency change model reveals that a lower *N* results in a higher sensitivity. Despite its lower sensitivity at *N* = 7, its lower operating frequency implies that high-performance circuits are not necessitated for measuring high frequencies, hence the tradeoff between sensitivity and operating frequency in terms of *N*.

Similar pH sensing experiments were performed to investigate the effect of *R_D_* on sensitivity (Figure 3c). Figure 3d shows that the sensitivity increased with *R_D_*. The oscillation frequency is a function of power effectively delivered by *V_ctrl_*, where *V_ctrl_* is obtained using the voltage divider circuit of the resistances of the load resistor and readout transistor. The voltage divider model of *V_ctrl_* can be expressed as*V_ctrl_* / *V_DD_* = *R_T_* / (*R_D_* + *R_T_*), where *R_T_* is a function of *I_D_* or *V_T_* of the readout transistor. Consequently, a higher *R_D_* results in a lower operation frequency. The effect of the load resistor on the sensitivity can be directly explained based on *V_ctrl_*. In other words, the gain of the readout is translated into the pH sensitivity. The small voltage variation due to the pH value multiplied by the transconductance *g_m_* of the readout transistor is the *I_D_* variation. According to Ohm’s law (*V* = *IR*), for the voltage divider of the load resistor and readout transistor, the *I_D_* variation multiplied by the load resistance yields *V_ctrl_* variation. Consequently, the gain of the readout circuit or sensitivity is *g_m_*⋅*R_D_*. A higher transconductance and a higher *R_D_* result in a more sensitive sensor. However, when *R_D_* is excessively large, the voltage drop across the load resistor can be dominant, and *V_ctrl_* becomes extremely low. In this case, the swing of *V_ctrl_* and the sensor resolution can be limited. In extreme cases, *V_ctrl_* can vary nonlinearly with pH because of the small swing.

Finally, the effect of *V_LG_* on sensitivity was analyzed, as shown in Figure 3e. Figure 3f shows that the sensitivity increases with the *V_LG_*. As explained earlier, the gain of the readout transistor or the sensitivity of the sensor is *g_m_*⋅*R_D_*, where *g_m_* increases with the DC gate bias of the readout transistor. However, if the DC gate bias increases significant, more *I_D_* will flow and the gain will be compromised by power consumption. This is particularly important if the sensor is intended for an always-on application. In addition, similar to the voltage swing of *V_ctrl_* being constrained by a high *R_D_*, a high *g_m_* yields the same result. Therefore, the tradeoff between sensitivity and nonlinearity/resolution/power consumption must be considered.

These experiments were consistent with an analytical equation that indicates the same dependence of the circuit parameters (*N*, *R_D_*, and *V_LG_*) on the frequency sensitivity (See Section 3.4):

$$\frac{df}{dpH}\propto \frac{(V_{LG}-V_{T})R_{D}}{N},$$

where *V_T_* is the threshold voltage of the readout transistor. Furthermore, the circuit simulations exhibited the same effects of the circuit parameters (*N*, *R_D_*, and *V_LG_*) on the frequency sensitivity (Figure S5). Understanding of signal amplification will be beneficial to the future design of highly sensitive frequency-based biosensors.

### 3.4. Analytical Equation for Investigating Effects of Circuit Parameters on Sensitivity

To increase the sensitivity of the sensor, we derived a theoretical equation to express the effect of each circuit parameter on the sensitivity. The sensitivity can be calculated by taking the derivative of the oscillation frequency (*f*) with respect to the pH, as follows:

(1) $$\frac{df}{dpH}=\frac{dV_{T}}{dpH}\frac{dV_{ctrl}}{dV_{T}}\frac{df}{dV_{ctrl}}=\alpha \frac{dV_{ctrl}}{dV_{T}}\frac{df}{dV_{ctrl}}$$

where *α* = *dV_T_ / dpH* is the pH sensitivity of the Al_2_O_3_ sensing layer caused by a pH-based surface potential change.

The drain current *I_D_* of the readout transistor biased in saturation is expressed as

(2) $$I_{D}=\frac{1}{2}\mu_{n}C_{ox}\frac{W}{L}{\left(V_{LG}-V_{T}\right)}^{2}=\frac{k}{2}{\left(V_{LG}-V_{T}\right)}^{2}$$

where *μ_n_* is the mobility of the electrons in the inversion layer, *C_ox_* the oxide capacitance, *W* the channel length, and *L* the channel width, and parameter *k* = *μ_n_C_ox_W* / *L* includes geometry and technology-dependent parameters.

When the drain current *I_D_* that flows through the load resistor *R_D_* causes a voltage drop across the resistor, the control voltage *V_ctrl_* can be expressed as

(3) $$V_{ctrl}=V_{DD}-I_{D}R_{D}=V_{DD}-\frac{k}{2}{\left(V_{LG}-V_{T}\right)}^{2}R_{D}$$

Subsequently, the second term in Equation (1) can be calculated by taking the derivative with respect to *V_T_* as follows:

(4) $$\frac{dV_{ctrl}}{dV_{T}}=k(V_{LG}-V_{T})R_{D}$$

The oscillation frequency *f* is expressed as

(5) $$f=\frac{1}{2Nt_{p}}$$

where *N* is the number of stages, and *t_p_* is the propagation delay per stage. The delay time *t_p_* is a function of the load capacitance (*C_L_*), supply voltage of the ring oscillator (*V_ctrl_*), and drain current (*I_Dsat_*) that flows through the ring oscillator, as follows [51]:

(6) $$t_{p}=\frac{C_{L}V_{ctrl}}{2I_{Dsat}}=\frac{C_{L}V_{ctrl}}{k{\left(V_{ctrl}-V_{T0}\right)}^{2}}$$

where *V_T0_* is the ring oscillator threshold voltage. It is noteworthy that the ring oscillator has the same initial threshold voltage as the readout transistor because the same n-type transistors (CD4007UBE, Texas Instruments, Dallas, TX, USA) are used. However, in contrast to *V_T_*, *V_T0_* is not a function of pH.

Substituting Equation (6) into Equation (5) yields

(7) $$f=\frac{1}{2N}\frac{k{\left(V_{ctrl}-V_{T0}\right)}^{2}}{C_{L}V_{ctrl}}$$

Subsequently, the third term in Equation (1) can be calculated by taking the derivative with respect to *V_ctrl_*:

(8) $$\frac{df}{dV_{ctrl}}=\frac{k}{2NC_{L}}\left(1-\frac{V_{T0}{}^{2}}{V_{ctrl}{}^{2}}\right)≃\frac{k}{2NC_{L}}$$

Finally, the sensitivity equation is obtained using Equations (1), (4), and (8) as follows:

(9) $$\frac{df}{dpH}=\alpha \frac{k^{2}}{2NC_{L}}(V_{LG}-V_{T})R_{D}$$

The sensitivity increases as the number of stages *N* decreases, the load resistance *R_D_* increases, and the liquid-gate voltage *V_LG_* increases.

### 3.5. pH Sensing Using Portable System

**Figure 4:**
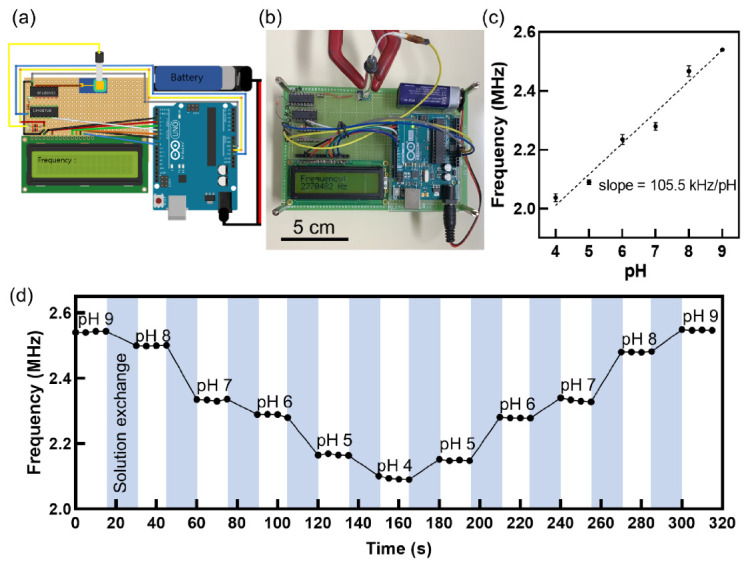
Portable measurement system. (**a**) Schematic illustration of portable measurement system. Small-sized Arduino board to replace bulk-sized oscilloscope for measuring oscillation frequency of sensor; 9 V battery supplies power to circuit. Operating conditions were *V_DD_* = 3.3 V, *V_LG_* = 2 V, *R_D_* = 1 kΩ, and *N* = 3. (**b**) Optical image of portable measurement system. Measured frequency displayed on the LCD screen. (**c**) Oscillation frequency vs. pH. (**d**) Real-time measurement of pH using portable measurement system.

A portable pH sensing system was constructed by replacing the bulky oscilloscope for the frequency measurement with a compact Arduino board, a general-purpose microprocessor capable of counting the number of oscillations via open-source codes (Figure 4a,b). Figure 4b shows that all the components for pH sensing, except the reference electrode, were integrated on a PCB board, on which the LCD screen was mounted to display the frequency measured according to the pH value of the test solution. The oscillation frequency in the portable sensing system increased with the pH value (Figure 4c), showing the same trend as that of the oscilloscope. The portable sensing system successfully demonstrated the real-time measurement of the pH response with high reproducibility (Figure 4d).

However, the portable sensing system requires futher developments for practical applications. First, the bulky Ag/AgCl reference electrode should be miniaturized to a compact size. Recent studies have demonstrated such miniaturization using an integrated on-chip gold gate [52] or an integrated fluidic chamber comprising an agarose gel sealed with a UV-curable resign [53], which is compatible with our sensor fabrication. Second, the accuracy of the frequency measurement of the portable sensing system should be further improved such at it can detect target analytes in a small dynamic range of interest [54]. Because the output waveform of the ring oscillator is not an ideal sinusoidal waveform (Figure 2a), some errors may be generated during frequency extraction; however, they will be rectified via noise reduction using a Schmitt trigger (Figure S6).

Wireless signal transmission can be simply and inexpensively implemented by connecting a Bluetooth module to the Arduino and using a smartphone application [55,56]. Through the Bluetooth connection between the Arduino and the smartphone, sensing data from the Arduino can be transferred to the smartphone screen instead of the LCD, which will be a subject of our further work (Figure S8). This platform not only receives sensing data in real time, but is also promising and versatile for POCT applications.

## 4. Conclusions

We demonstrated a conversion method that enables surface potential-controlled oscillations to provide the oscillation frequency as an output parameter for FET-based biosensors. Unlike conventional parameters such as *V_T_* and *I_D_*, the oscillation frequency requires neither bulky and expensive analysis equipment nor complicated and specified ICs. However, it is compatible with open-source electronics platforms such as the Arduino board. The ring oscillator connected to the extended-gate biosensor can convert the pH-induced surface potential generated in the Al_2_O_3_ layer of the extended gate into an oscillation signal. The proposed sensor demonstrated sensitive and reliable pH sensing based on the linear dependence of the oscillation frequency on pH, suggesting the usefulness of the oscillation frequency for the analysis of FET-based biosensors. Our comparison of the *V_T_* signal and oscillation frequency revealed that they were directly correlated with each other as well as with the surface potential, and that the sensing electrode that can produce a higher surface potential was key for signal amplification. The pH sensing experiments, analytical calculations, and circuit simulations confirmed that the frequency signal upon the pH response can be further amplified by controlling the circuit parameters, such as by reducing *N*, increasing *R_D_*, and increasing *V_LG_*. A portable sensing system for real-time pH measurement was constructed on a PCB board by integrating an extended-gate biosensor and an Arduino board to measure the frequency signal. The surface potential-controlled oscillation in this study is suitable for not only POCT applications, but also neuromorphic applications based on spiking neural networks.

## Data Availability

All data collected in this study are presented within this article and Supplementary Materials.

## Supplementary Materials

The following are available online at https://www.mdpi.com/1424-8220/21/6/1939/s1, Figure S1: Optical microscopy images of sensing electrode, Figure S2: pH sensing characteristics of extended-gate FET with Al_2_O_3_ layer, Figure S3: Comparison between threshold voltage and oscillation frequency, Figure S4: Conversion of gate voltage to oscillation frequency, Figure S5: PSpice simulations for investigating effects of circuit parameters on sensitivity, Figure S6: Comparison between performances of oscilloscope and Arduino, Figure S7: Arduino board-pin mapping, Arduino source codes for frequency measurement, and Figure S8: Wireless signal transmission.
