# Supplementary material for: Surface Potential-Controlled Oscillation in FET-Based Biosensors
Source: Sensors (Basel). 2021 Mar 10;21(6):1939. doi: 10.3390/s21061939 (PMC8061884; doi:10.3390/s21061939)
Supplement: Supplementary file 1 [file sensors-21-01939-s001.pdf]

Supplementary Materials

Surface potential-controlled oscillation in FET-based biosensors

Ji Hyun Kim[1,†], Seong Jun Park[1,†], Jin-Woo Han[2], Jae-Hyuk Ahn[3,*]

[1] Department of Electronic Engineering, Kwangwoon University, Seoul 01897, Korea,

[2] Center for Nanotechnology, NASA Ames Research Center, Mountain View, CA 94035, USA,

[3] Department of Electronics Engineering, Chungnam National University, Daejeon 34134, Korea

[*] Correspondence to J.-H. Ahn (E-mail: jaehyuk@cnu.ac.kr)

[†] These authors contributed equally to this work.

Table of contents:

S1. Optical microscopy images of sensing electrode

S2. pH sensing characteristics of extended-gate FET with Al_2O_3 layer

S3. Comparison between threshold voltage and oscillation frequency

S4. Conversion of gate voltage to oscillation frequency

S5. PSpice simulations for investigating effects of circuit parameters the sensitivity

S6. Comparison between performances of oscilloscope and Arduino

S7. Arduino board-pin mapping

S8. Arduino source codes for frequency measurement

S9. Wireless signal transmission

S1. Optical microscopy images of sensing electrode

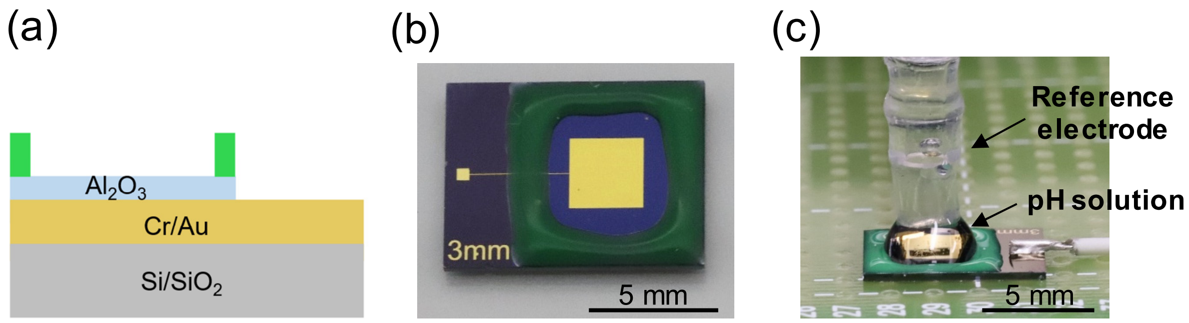

Figure S1. (a) Schematic illustration of Al_2O_3-deposited metal electrode ($Cr/Au/Al_2O_3$ = 3 nm/100 nm/15 nm). (b, c) Sensing electrodes with reservoir produced using silicone elastomer to contain test solution.

S2. pH sensing characteristics of extended-gate FET with Al_2O_3 layer

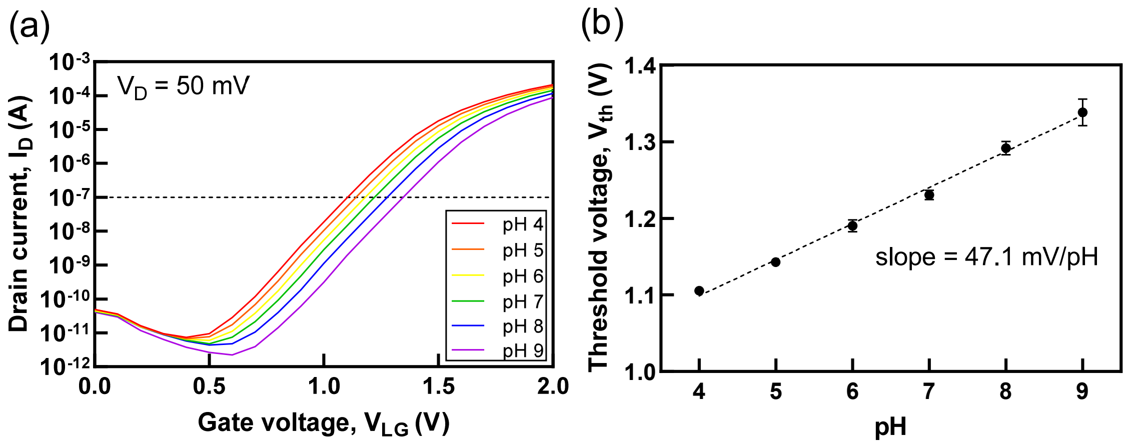

Figure S2. (a) Drain current (I_D)–liquid-gate voltage (V_{LG}) characteristics of Al_2O_3-deposited extended-gate FET as a function of pH. (b) Threshold voltage of extended-gate FET vs. pH.

S3. Comparison between threshold voltage and oscillation frequency

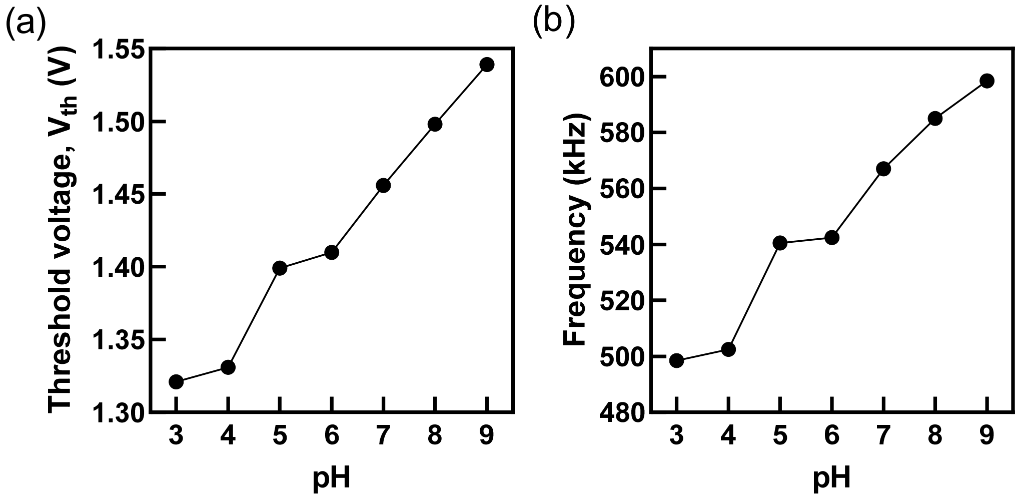

Figure S3. (a) Threshold voltage vs. pH and (b) oscillation frequency vs. pH, measured on the same Al_2O_3 sensing electrode under conditions of N = 3, V_{DD} = 2 V, V_{LG} = 1.5 V, and R_D = 1 kΩ.

S4. Conversion of gate voltage to oscillation frequency

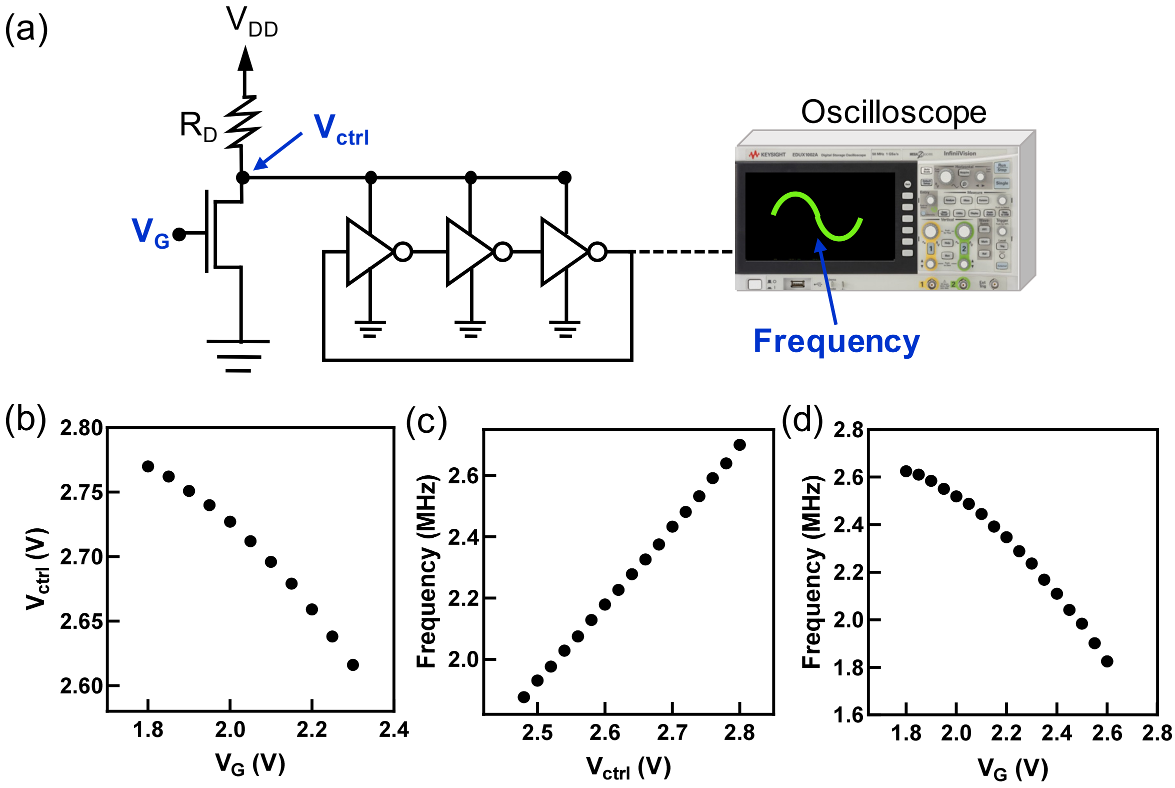

Figure S4. (a) Frequency measurement setup. (b) V_G vs. V_{ctrl}. (c) V_{ctrl} vs. oscillation frequency. (d) V_G vs. oscillation frequency. Operating conditions were V_{DD} = 3.3 V and R_D = 1 kΩ.

S5. PSpice simulations for investigating effects of circuit parameters on sensitivity

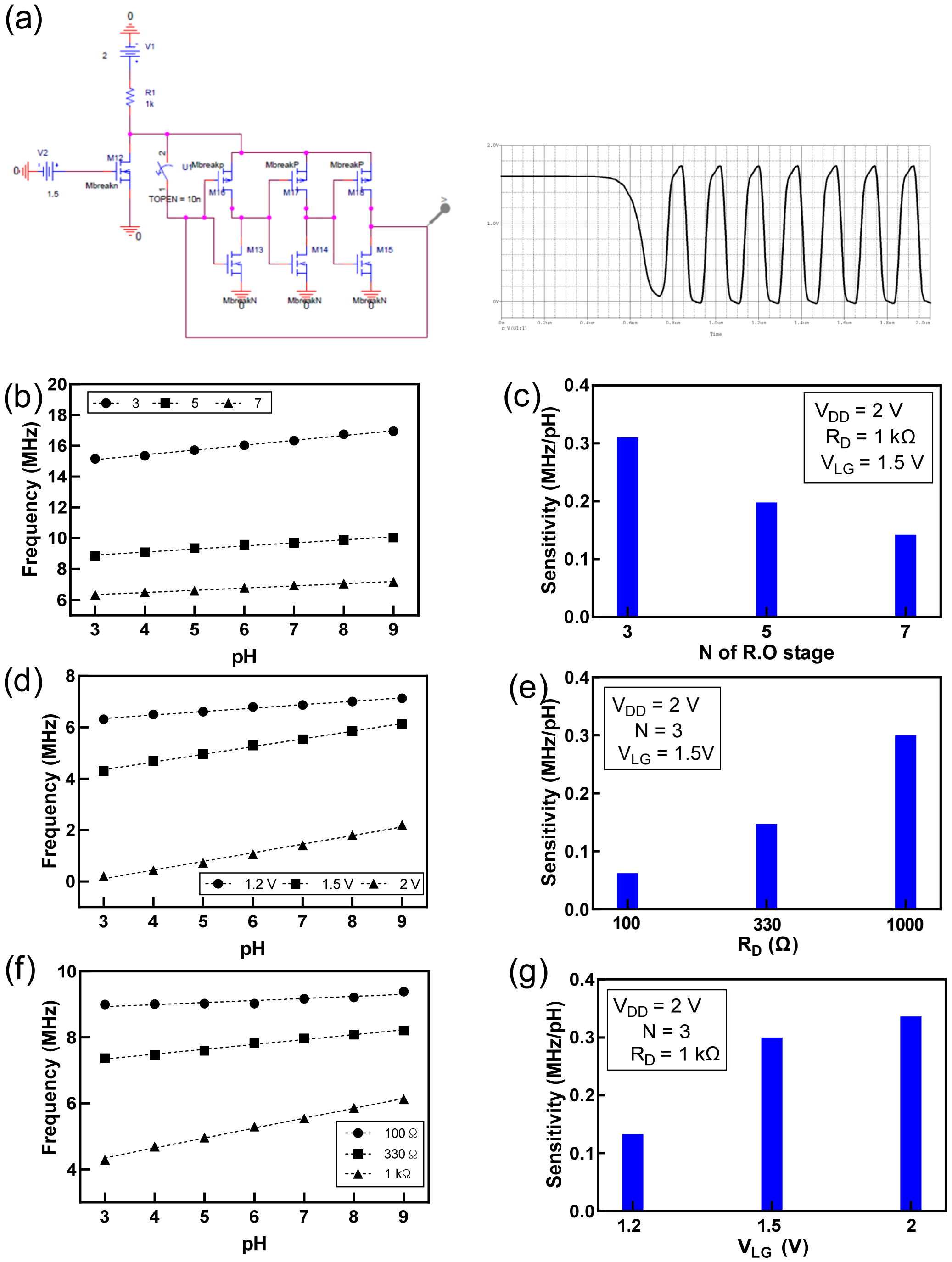

Figure S5. (a) Circuit diagram for PSpice simulation and typical output waveform. (b) Oscillation frequency vs. pH for different numbers of stages (*N*). (c) Dependence of sensitivity on *N*. (d) Oscillation frequency vs. pH for different values of drain resistance (R_D). (e) Dependence of sensitivity on R_D. (f) Oscillation frequency vs. pH for different values of liquid-gate voltage (V_{LG}). (g) Dependence of sensitivity on V_{LG}.

S6. Comparison between performances of oscilloscope and Arduino

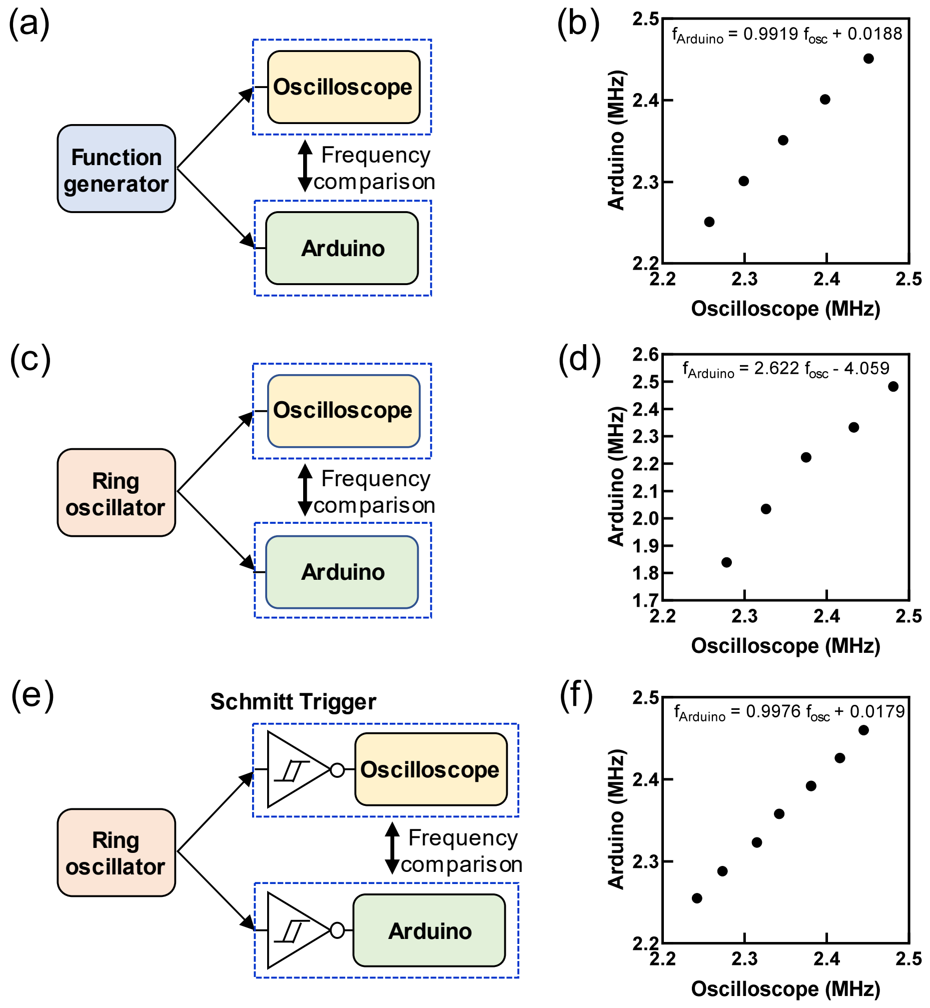

Figure S6. (a, b) Comparison of frequencies measured using oscilloscope (EDUX1002A, Keysight) and Arduino board (Arduino UNO R3) for frequency generated by function generator (33210A, Keysight). (c, d) Comparison of frequencies measured using oscilloscope and Arduino board for frequency generated by three-stage ring oscillator (CD4007UBE, Texas Instruments). (e, f) Comparison of frequencies measured using oscilloscope and Arduino board connected with Schmitt trigger (74LS14N, Sanken) for frequency generated by three-stage ring oscillator (CD4007UBE, Texas Instruments).

S7. Arduino board-pin mapping

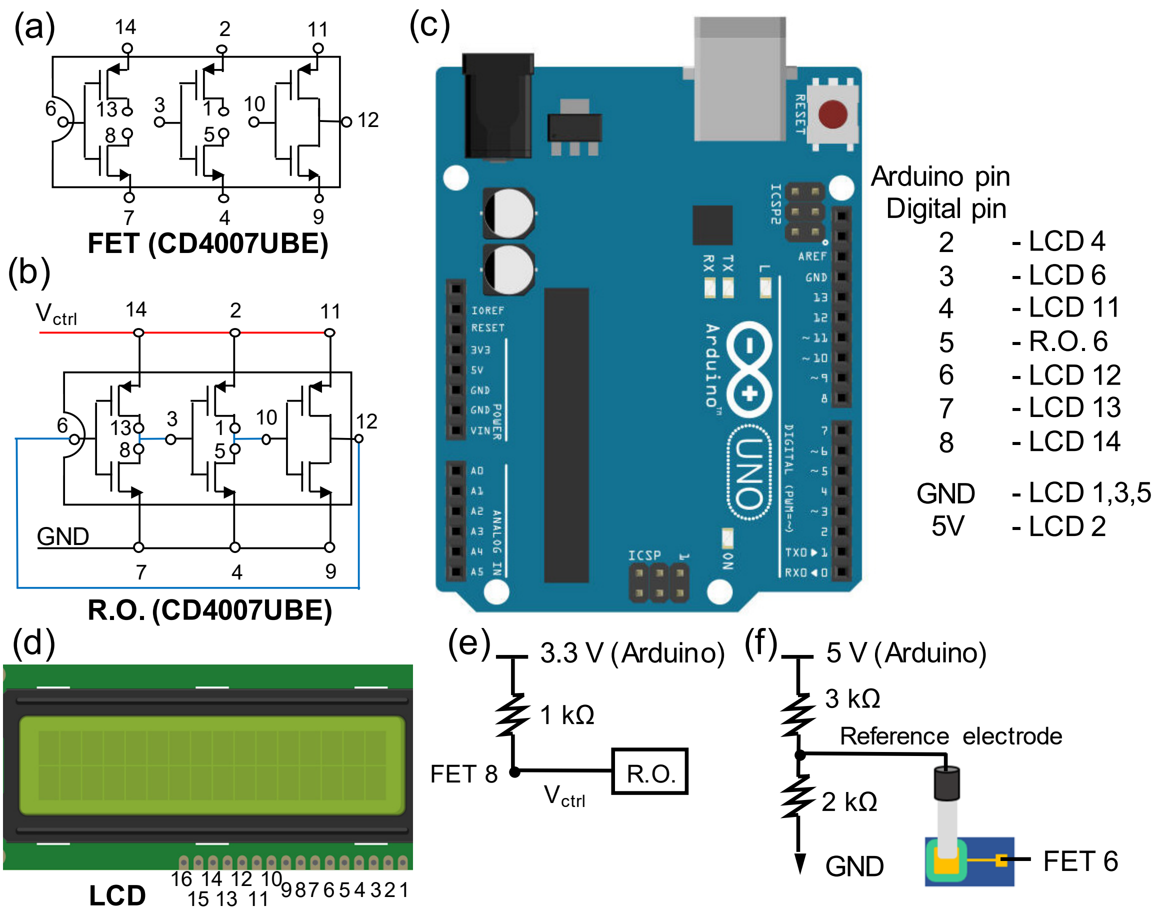

Figure S7. (a) Schematic diagram of CD4007UBE for readout transistor. (b) Ring oscillator arrangement using CD4007UBE. (c) Arduino Uno R3 pinout. (d) LCD module (SZH-EK101, SMG) pinout. (e) Connection of readout transistor to 3.3-V supply voltage. (f) Voltage divider circuit for applying liquid-gate voltage to extended gate through reference electrode.

S8. Arduino source codes for frequency measurement [S1]

```
#include <FreqCount.h>
#include <LiquidCrystal.h>        // include Arduino LCD library

// LCD module connections (RS, E, D4, D5, D6, D7)
LiquidCrystal lcd(2, 3, 4, 6, 7, 8);

void setup(void) {
  // set up the LCD's number of columns and rows
  lcd.begin(16, 2);
  lcd.print("Frequency:");
  // initialize freqCount library with time basis of 1000ms (1 second)
  // Arduino counts number of pulses during period of 1 second
  FreqCount.begin(1000);

}
// main loop
void loop() {

  if (FreqCount.available()) {
    unsigned long count = FreqCount.read();

    lcd.setCursor(0, 1);
    lcd.print(count);    // print frequency value in Hz
    lcd.print(" Hz           ");
  }

}
```

S9. Wireless signal transmission

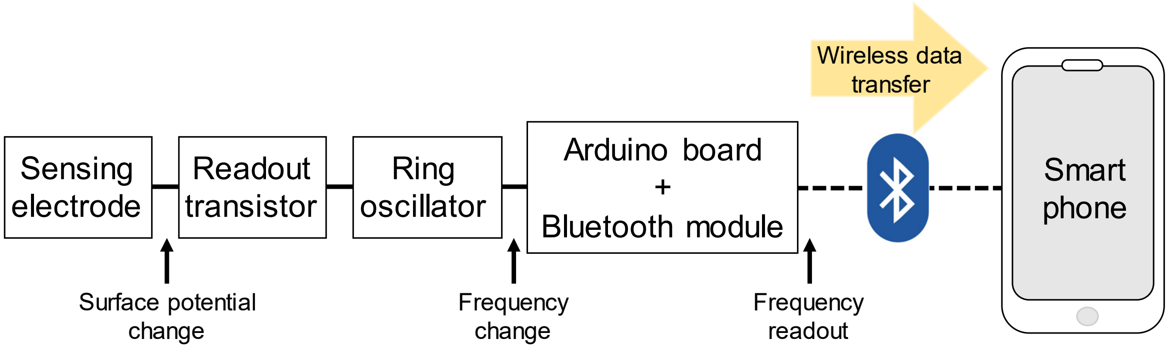

Figure S8. Schematic diagram for wireless sensor integration.

References

S1. Simple Electrical and Electronics Projects Home page. Available online: https://simple-circuit.com/arduino-frequency-counter-project/ (accessed on 2 February 2021)
